# Human Umbilical Cord Mesenchymal Stem Cell-derived Exosomes Induce Macrophage M2 Polarization by Antagonizing LPS-mediated Stimulation of the NF-κB and STAT3 Pathways

**DOI:** 10.2174/0113862073314685240514050119

**Published:** 2024-05-15

**Authors:** HengJin Tian, AMin Chen, PeiYao Gao, FeiFan Wang, YanMing Zhao, FengChao Wang, Chaoqun Lian, Qiang Zhang

**Affiliations:** 1 Department of Clinical Laboratory, The First Affiliated Hospital of Bengbu Medical University, Bengbu, 233004, China;; 2 Key Laboratory of Cancer Research and Clinical Laboratory Diagnosis, Bengbu Medical university, Bengbu, 233030, China;; 3 Department of Blood Transfusion, The First Affiliated Hospital of Naval Medical University, Shanghai, China;; 4 Department of Clinical Laboratory, The Second People's Hospital of Bengbu, Bengbu, 233000, China

**Keywords:** Mesenchymal stem cells (MSCs), human umbilical cord MSCs (hucMSCs), exosomes, macrophage, polarization, anti-inflamaeory

## Abstract

**Background:**

Many studies have documented the protective effects of regulating macrophage M1/M2 polarization in inflammatory diseases characterized by their imbalance state. In pathological diseases associated with inflammation, mesenchymal stem cells (MSCs) regulate macrophages, thereby having anti-inflammatory and tissue regenerative effects. Exosomes have been suggested as an alternative mechanism that underlies the paracrine function of MSCs. Thus, this study explored the anti-inflammatory impact of human umbilical cord MSCs-secreted exosomes (hucMSCs-EX) by influencing macrophage polarization in normal and inflammatory environments *in vitro*.

**Methods:**

In this study, hucMSCs-conditioned medium (hucMSCs-CM) and hucMSCs-EX were used to treat RAW264.7 macrophages with or without LPS. The expressions of *TNF-α, IL-10, IL-6, IL-1β*, and *Arg-1* were quantified by qPCR. The expressions of *IL-6* and *IL-10* were evaluated by ELISAs. Western blots (WB) were performed to observe the expressions of CD206, NF-κB P65, NF-κB p-p65, p-STAT3, STAT3, and NF-κB phosphorylation. The number of cells expressing CD206 and the fluorescence intensity were measured *via* flow cytometry (FC) and immunofluorescence staining. Cell propagation and migration were examined *via* MTT and transwell assays, respectively.

**Results:**

The inhibition of LPS-induced inflammatory polarization by hucMSCs-EX or hucMSCs-CM led to increases in *IL-10* and *arginase* (Arg) levels and decreases in those of *IL-6* and *TNF-α*. Moreover, hucMSCs-EX enhanced the CD206 expression in RAW264.7 cells and accelerated the propagation and migration of LPS-induced cells. The suppressive impact of hucMSCs-EX on the LPS-induced phenotypic polarization of M1 macrophages was linked with the reduction of NF-κB signaling. They stimulated the transition of M2 macrophages by enhancing the activity of STAT3 in RAW264.7 cells.

**Conclusion:**

This study indicated that hucMSCs-EX enhances the macrophage transition into the M2 phenotype by inhibiting the NF-κB p65 axis and stimulating that of STAT3.

## 1 INTRODUCTION

Macrophages have a crucial role in the immune responses against several clinical diseases, such as viral and autoimmune diseases [1, 2]. In tissues, circulating blood monocytes differentiate into macrophages and remain distinct phenotypic changes that correspond to the different phases of inflammation, namely, M1 macrophages that are classically activated and promote inflammation and M2 macrophages that are alternatively activated [3]. Moreover, M1 and M2 macrophages can interconvert under different environmental conditions. There is growing evidence indicating that M1 macrophages predominate in the blood vessels around inflammatory wound tissues. They can be recruited by Interferon-γ (IFN-γ)/LPS to secrete cytokines such as *IL-1β, IL-6, TNF-α* and *IFN-γ.* Moreover, M2 macrophages hold predominantly anti-inflammatory characteristics and have a role in the alleviation of inflammation [4]. They substantially suppress inflammation by upregulation of *Arg-1* and *IL-10*. They also participate in tissue remodeling and angiogenesis [5]. Plasticity and diversity are two of the most distinguishing features of macrophages. The various polarization phenotypes of macrophages have a crucial role in maintaining homeostasis and influencing the development, progression, and other stages of inflammation [6, 7]. Therefore, directing the macrophage polarization towards either the M1 or M2 phenotype has appeared as a prospective therapeutic option for many inflammatory conditions [5, 8].

Multiple studies have revealed the possible involvement of mesenchymal stem cells (MSCs) in reducing inflammation, regulating the responses of immune cells, and aiding in the healing of different types of tissue injuries [9]. However, their application in clinical settings is constrained due to the tumorigenic characteristics of transplanted cells. Research has indicated that the therapeutic effects of MSCs primarily rely on their ability to secrete specific mediators *via* paracrine secretions, including lipids, proteins, mRNAs, and microRNAs encapsulated within exosomes [10]. MSCs-derived bio-active molecules/exosomes are closely interacted with immunocytes such as natural killer cells (NKCs), dendritic cells (DCs), lymphocytes, and macrophages. Exosomes are extracellular membrane vesicles (40 to 100 nm) that are secreted into the extracellular environment by multiple cell types, for instance, mast cells, DCs, reticulocytes, B and T cells, epithelial cells, MSCs, and tumor cells [11]. Once released, exosomes may enter biological fluids to facilitate the long-distance exchange of biological information mediated by exosomes [12]. Alternatively, they may remain close to their secretory cells. The cellular origin of exosomes dictates their composition [11]. Despite their source, exosomes contain several prominent proteins, including Alix3, CD9, and CD63 [13, 14]. Exosomes are important in local cell-to-cell communication and genetic information exchange by transferring membrane and cytosolic proteins, lipids, coding RNA, noncoding RNA, antigen-presenting molecules, and DNA between cells in the inflammatory microenvironment. Thus, they have the potential to influence the outcome of inflamed tissues [15, 16]. This study investigated the potential signaling pathways in which hucMSCs influence LPS-induced mouse RAW264.7 cells *via* their secreted exosomes. Results showed that hucMSCs-EX-co-cultured macrophages that were stimulated by LPS, were identified by reductions in *TNF-α* and *IL-6* and increases in *IL-10* [17] and *Arg-1*. Moreover, LPS-induced cells displayed enhanced proliferation and migration, and hucMSCs-EX upregulated the CD206 expression, a surface marker for the M2 macrophages. Importantly, the suppressive effect of hucMSCs-EX on LPS-induced phenotypic M1 polarization of macrophages was linked with the decreased level of the NF-κB pathway. In contrast, hucMSCs-EX-induced M2 macrophage polarization is associated with STAT3 pathway activation in RAW264.7 cells. The current study validates the promising prospects of hucMSCs-EX as a clinically viable anti-inflammatory therapy by modulating macrophage polarization.

## 2 MATERIALS AND METHODS

### 2.1 Extraction, Purification, and Confirmation of hucMSCs-EX

Previous methods were followed for the isolation and propagation of hucMSCs [18]. These cells (third passage) were differentiated into osteoblasts and adipocytes using respective induction media (Gibco Grand Island, NY, USA). Alizarin Red and Oil Red O stains were used to observe osteogenic and adipogenic differentiation. Exosomes (hucMSCs-EX) derived from a conditioned medium of hucMSCs (hucMSCs-CM) were isolated and purified as per the established protocols [19]. Briefly, hucMSCs were grown to 60% confluence, and were rinsed three times with PBS to remove any particle of fetal bovine serum (FBS, Invitrogen). Cells were continuously grown without serum and exosomes in RPMI-1640 medium (Invitrogen) for 48 h. This exhausted medium is considered a conditioned medium derived from hucMSCs (hucMSCs-CM). The medium was collected and distributed into two parts: one part of hucMSCs-CM was filtered *via* a 0.22 μm syringe filter (Millipore, USA) and kept at -80°C for further use. The second part of hucMSCs-CM was centrifuged (1000 × g, 20 min) to discard cell debris, followed by two re-centrifugations (2,000 × g and 10,000 × g, 20 min each). The supernatants were purified (100 kDa MWCO [Millipore, USA], 1,000 × g, 30 min). The purified mixture was placed on a cushion of 5 mL of 30% sucrose/D2O and ultracentrifuged using a Beckman Coulter Optima L-90K ultracentrifuge (100,000 × g, 90 min, 4°C) [11]. The fractions enriched with microvesicles were obtained and then mixed with PBS. This was followed by three centrifugations (1,000 × g, 30 min) with 100 kDa MWCO. Precipitates were resuspended in sterile PBS and centrifuged (3000 rpm, 10 min, 4°C). After adsorption of 20 μL of the purified hucMSCs-EX onto copper grids and incubation at 25°C for two min, the grids were stained for 10 min with 30 μg/L phosphotungstic acid (pH 6.8) at 25°C [11]. The sample was dried at 25°C and then examined *via* transmission electron microscope (TEM) (Olympus, Tokyo, Japan). The size and dispersion of hucMSCs-EX were quantified and examined using Nanosight NTA and the Zetasizer-Nano software. Finally, the purified hucMSCs-EX were extracted, filtered (0.22 μm), and then kept at -80°C for further use. Bichicondinic acid (BCA) was employed to determine the protein concentration of the isolated exosomes. The resulting value was then used to calculate the exosomes' concentration. Approximately 50 μg/mL exosomes were preserved at -80°C until use. Fresh umbilical cord tissues were collected from full-term newborn delivered through cesarean section after obtaining written consent from their parents [18]. The Ethics Committee of Bengbu Medical University, Bengbu, China, approved all the relevant experimental protocols.

### 2.2 Co-culture of Macrophages (RAW264.7) with hucMSCs-EX

The cell line (murine macrophage RAW264.7) was received from the Cell Bank of Shanghai, China. Cells in logarithmic growth were grown in RPMI-1640 with 10% FBS in the presence or absence of LPS (1 μg/mL) (5164948, Hangzhou, China) for 4 h. After incubation, cells were rinsed with PBS (thrice) and maintained in a complete medium (control), hucMSCs-CM (1:1 diluted while using a fresh medium), and 5 μg/mL hucMSCs-EX for 48 h in a 6-well dish. The propagated macrophages and resultant supernatants were obtained and stored for further experiments.

### 2.3 Gene Profile *via* qRT-PCR

Total RNA contents were isolated from respective cells *via* TRIzol reagent (Invitrogen). The cDNAs were reverse-transcribed as per the manufacturer's directions *via* a reverse transcription kit (Invitrogen). The qPCR reactions were conducted using primers that were specific to mice and targeted the *TNF-α*, *IL-10, IL-6, IL-1β, Arg-1,* and *β-actin* genes (Table **1**). mRNA expression was normalized with *β-actin*, which is regarded as the internal or standard control. The Bio-Rad Real-Time System was used to conduct PCR reactions *via* TB GreenTM Premix Ex TaqTM II (Tli RNaseH Plus).

### 2.4 Detection of cytokines *via* ELISA

As per the manual's instructions (Dakewe, Beijing, China), the quantity of IL-6 and IL-10 in the collected supernatants was monitored using ELISA kits. The absorbance (OD) was detected at 450 nm *via* the NanoDrop™ 8000 Spectrophotometer (ND-8000-GL, Thermo Scientific™).

#### 2.4.1 Flow Cytometry

Cells were grown in 6-well plates in appropriate media for 24 h. Next, the cells were labeled with anti-CD206 antibody (1:20, ARG22456, Arigobio, China) at 25°C for 30 min and then kept with FITC-labeled secondary antibody (ARG23840, 1:500, Arigobio) at 25°C for 15 min. The proportion of cells expressing CD206 was measured by FC.

### 2.5 Western Blot Analysis

The lysates of total or nuclear proteins were isolated from cultured cells and hucMSCs-EX. Proteins contents were quantified with a BCA kit (Thermo Fisher Scientific). Protein in aliquots of equal weight (40 μg) was loaded onto a 10% SDS-PAGE and subsequently transferred to nitrocellulose membranes. After 1 h of blocking in 5% skim milk at 25°C, the blots were kept at 4°C for 24 h with respective primary antibody dilutions and then labeled with secondary HRP-linked antibody at 37°C for 2 h. The bands of protein were detected *via* an HRP substrate (EMD Millipore). and examined using MD ImageQuantTM Software (G: Box; Syngene, Cambridge, UK). β-actin, as well as histone H3.1, were employed as reference controls for cell cytoplasmic and nuclear proteins. Following were the Rabbit monoclonal antibodies used for WB analysis: anti-human CD9 antibody (1:500: BS60359, Bioworld Technology, USA), anti-human CD63 (1:1000: ab134045, Abcam, Cambridge, UK), anti-human Alix 3 (1:1000: ab186728, Abcam), anti-Lamin A antibody (1:2000: ab108595, Abcam), anti-human β-Tubulin (1:2000: AP0064, Bioworld), anti-mouse CD206 (1:1000: ab64693, Abcam), anti-mouse NF-κB p65 (1:1000: #C48676, SAB, CA), anti-mouse NF-κB p-p65 (S536, 1:1000: ab76302, Abcam), anti-mouse p-STAT3 (Tyr539, 1:500, #13150, SAB), STAT3 (1:500, #53229, SAB), Rabbit polyclonal anti-mouse Histone H3.1 (1:500: BS90644(P68433), Bioworld) and anti-β-actin (1:2000: AP0060, Bioworld). The goat anti-Rabbit IgG(H+L)-HRP antibody (1:2000, Bioworld Technology Cat# BS13278, RRID: AB_2773728) was purchased from Bioworld.

### 2.6 Immunofluorescence Staining

Cells were cultured for 24 h on glass slides coated in 24-well plates. Further, they were co-incubated with complete RPMI-1640 medium, hucMSCs-CM, and hucMSCs-EX for 48 h. Cells were rinsed twice with PBS and fixed in 4% paraformaldehyde (PFA, AR-0211; DingGuo Biotechnology China) solution for 30 min at 4°C. Blocking (5% BSA) was performed and cells were kept with rabbit anti-mouse CD206 monoclonal antibody (1:500, ARG22456, Arigobio) at 4°C for 24 h and followed by FITC-linked anti-rabbit secondary antibodies (1:200, ARG23840, Arigobio) at 37°C for 2 h. Cells were stained with DAPI to detect cell nuclei. Fluorescence images of cells were obtained using ECLIPSE Ti-S, Nikon, Japan.

### 2.7 Cell Proliferation Assays

Metabolic or proliferative activity of RAW264.7 cells was detected *via* the MTT assay. Precisely, approximately 2 × 103 cells/well were grown in a 96-well plate and kept with 100 μL of complete medium (serve as the control group), hucMSCs-CM and hucMSCs-EX mixed in the presence or absence of LPS (1 μg/mL). The cells were subjected to incubation at 25°C for 4 h after adding 20 μL MTT to each well at the respective times of 24, 48, and 72 h. Finally, the media were discarded and absorbances were monitored at 450 nm *via* a microplate reader (RNE90002, USA).

### 2.8 Cell Migration Assays

In this assay, RAW264.7 cells (5 × 104 in 200 μL of incomplete medium) were allowed to grow in a 24-well plate (8.0-µm polycarbonate membrane) (Costar, CA, USA). They were seeded to the upper surface of the compartment while the lower surface was filled with only medium (600 µL). Further, hucMSCs-CM and 600 µL hucMSCs-EX were mixed, respectively. In each well, LPS (1 μg/mL) was added. The upper surface of the membrane was cleared of any residual cells after a 12-h incubation *via* a cotton swab. The migrated cells were fixed for 8 min with 4% PFA on the lower surface of the membrane. Crystal violet solution was used to stain the cells for 15 min. After washing with PBS (thrice) cells were dried, examined, and quantified under an inverted microscope (Olympus, Tokyo).

### 2.9 Statistical Analysis

Data was statistically evaluated *via* GraphPad 9.3 (Graph Pad Software) and SPSS 21.0 (IBM Corp.) software and were depicted as mean ± SEM derived from ≤ triplicate analyses. Group comparison was performed using Student’s t-test and One-way ANOVA with Tukey’s post hoc tests. A p-value ≤ 0.05 was regarded as a significant threshold.

### 2.10 Ethical Considerations

This study was performed on cell lines and on human umbilical cord tissue. Human umbilical cords used in this study are normally discarded as medical waste so no ethical concerns are involved in obtaining hucMSCs-EX. There were no humans were directly involved in this study. No work with human subjects was directly involved in our study.

## 3 RESULTS

### 3.1 Characterization of hucMSCs and hucMSCs-EX

**Fig. (1):**
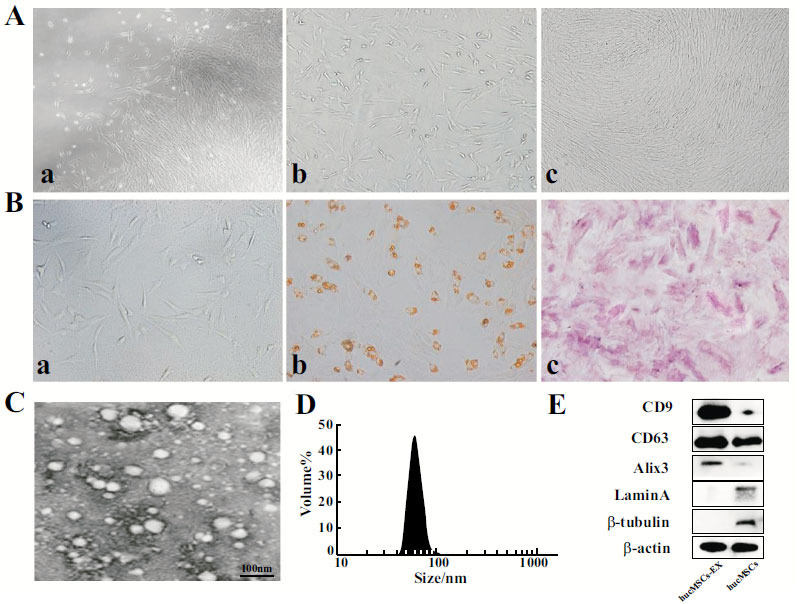
**Identification of hucMSCs and hucMSCs-EX. (A)** Images of the hucMSCs' morphology under a light microscope. (1) Morphology of primary hucMSCs (100x);(2, 3) Morphology of hucMSCs at third passage (100x). **(B)** Identification of differentiation potential of hucMSCs *in vitro*. (1) hucMSCs of the third passage serve as a control; (2, 3) Differentiation of hucMSCs was assessed using Alizarin Red and Oil Red O stains. **(C)** Exosomes derived from hucMSCs (hucMSCs-EX) are depicted in TEM images. Scale bar 100 nm. **(D)** Nanosight Particle Size Analyzer was used to detect the particle size distribution of hucMSCs-EX. **E,** Representative WB analysis for exosome proteins (CD63, CD9, and Alix3). Triplicate experiments were carried out. hucMSCs are abbreviated for human umbilical cord MSCs.

Primary cultured hucMSCs adhered to the plastic surface after migrating from the human umbilical cord tissues. They have fibrous morphology and fingerprint-like growth characteristics under an inverted microscope (Fig. **1A1** and **A2**). In the third passage, after subcultures, a population of hucMSCs that was relatively homogeneous was observed (Fig. **1A3**). Multidirectional *in vitro* differentiation (adipogenic and osteogenic) was displayed by hucMSCs. As shown in Fig **1B2**, the cells comprised fat particles and were stained red; similarly, calcium deposits were stained red in the cells (Fig. **1B3**). Exosomes were predominantly round lipid-coated microvesicles resembling tea saucers, as detected using TEM. The exosomes showed a range of sizes from 40 to 100 nm (Fig. **1C** and **D**). The Western blot (WB) analysis revealed an upregulation of exosome proteins CD9, CD63, and Alix3 in the hucMSCs-EX (Fig. **1E**). The extraction of hucMSCs-EX was successful.

### 3.2 *In vitro*, hucMSCs-EX Stimulated M2 Macrophage Polarization

**Fig. (2):**
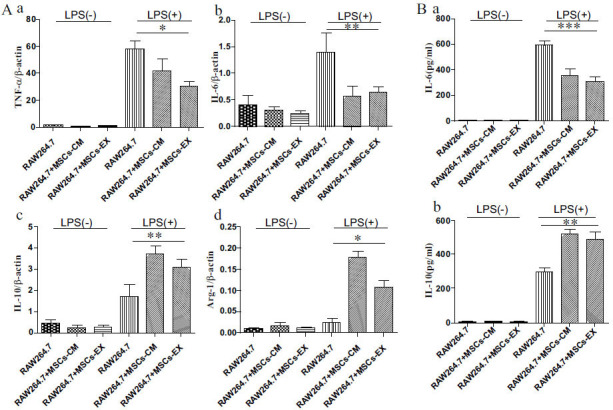
hucMSCs-EX increased M2 macrophage gene expression and decreased M1 gene expression in LPS-stimulated cells. **(A),** In the experiment, RAW264.7 cells were treated with LPS (1μg/mL) for 4 h. They were incubated with a complete medium, hucMSCs-CM (1:1 in fresh complete medium), and 5μg/mL hucMSCs-EX for 48 h. The mRNA levels of *TNF-α* (**a**), *IL-6* (**b**), *IL-10* (**c**), and *Arg-1* (**d**) were quantified using qRT-PCR. **(B)** The expressions of *IL-6* (**a**) and *IL-10* (**b**) in the collected supernatant, as described above, were determined *via* ELISA (*p ≤ 0.05, ** *p* ≤ 0.01, *** *p* ≤ 0.001).

Compared with RPMI-1640 medium, hucMSCs-CM or hucMSCs-EX significantly increased *Arg-1* and *IL-10*, while markedly reducing *TNF-α* and *IL-6* expression in LPS-induced cells (Fig. **2A**). Further, *IL-6* and *IL-10* were identified in the culture supernatants of the macrophages *via* ELISA, as previously outlined. In line with the qRT-PCR findings, hucMSCs-EX or hucMSCs-CM substantially inhibited the secretion of *IL-6* (Fig. **2Ba**), an essential pro-inflammatory cytokine, while markedly increasing the release of IL-10 (Fig. **2Bb**).

### 3.3 hucMSCs-EX Enhanced CD206 Expression

**Fig. (3):**
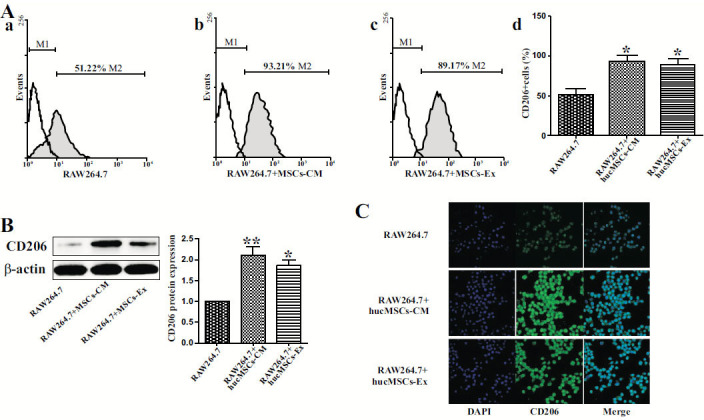
hucMSCs-EX enhanced CD206 expression. **(A),** Flow cytometric analysis displayed the number of CD206-positive cells. Cells were co-incubated with different media for 48 h: RPMI-1640 medium as a control group, (**a**) hucMSCs-CM, and (**b**) hucMSCs-EX (5μg/mL). Cells were then treated with (**c**) CD206 primary and FITC-conjugated secondary antibodies (d) Flow cytometry also analyzed the number of CD206-positive cells, and triplicate experiments were carried out for final analysis. **(B),** Isolated proteins from RAW264.7 cells stimulated with hucMSCs-EX were analyzed using WB with a particular antibody targeting CD206. **(C),** RAW264.7 cells were untreated or maintained with hucMSCs-EX for 48 h. Immunofluorescent staining monitored the levels of CD206 in respective cells *p ≤ 0.05, **p ≤ 0.01 vs. RAW264.7 (Control).

The protein expression of CD206 (mannose receptor), a widely known marker for M2 macrophages was detected to identify whether hucMSCs-EX elicited the M2 phenotype [20], in cells *via* FC and WB analysis. The hucMSCs-EX or hucMSCs-CM substantially raised the quantity of CD206 expressing macrophages (Fig. **3A**). In consensus with the findings from FC, immunofluorescence staining and WB analyses revealed substantially increased levels of CD206 in respective cells exposed with hucMSCs-CM and hucMSCs-EX, relative to the control group (Fig. **3B** and **C**).

### 3.4 hucMSCs-EX Stimulate RAW264.7 Cell Propagation and Migration

**Fig. (4):**
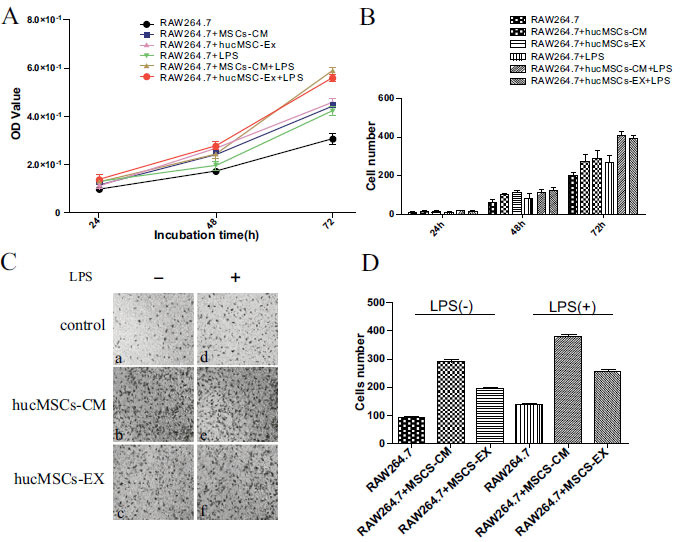
hucMSCs-EX enhanced LPS-stimulated RAW264.7 cell propagation and migration. **(A),** hucMSCs-EX enhanced the propagation of LPS-stimulated cells. Cells were cultured with or without LPS (1μg/mL) in complete medium, hucMSCs-CM and hucMSCs-EX for 24, 48, and 72 h, respectively. **(B),** Quantification of cell proliferation by cell counting assays **(C),** hucMSCs-EX promotes the migration of LPS-stimulated cells. Cells were inoculated on the upper surface of the chamber, and the bottom chambers were enriched with RPMI-1640 medium (**a**) or with LPS (**d**), hucMSCs-CM (**b**) or with LPS (**e**), and hucMSCs-EX (**c**) or with LPS (**f**), respectively. Representative images depict the invasion of cells treated with hucMSCs-EX. **(D),** the numbers of the migrated cells were counted after 12 h. (* *p* ≤ 0.05, ** *p* ≤ 0.01 *vs*. RAW264.7).

To identify whether hucMSCs-EX or hucMSCs-CM can promote macrophage proliferation and migration, MTT and cell counting assays were carried out. As depicted in Fig. (**4A**), the OD values of hucMSCs-EX and hucMSCs-CM groups were higher than those in the control and LPS-induced RAW264.7 groups 48 h after treatment, respectively. In line with the findings of the MTT, the cell counting assay demonstrated that hucMSCs-EX or hucMSCs-CM markedly enhanced proliferation in LPS-induced cells 48 h after treatment (Fig. **4B**). The migratory capability of respective cells after treatment with hucMSCs-EX was then evaluated. The data depicted in Fig (**4C** and **D**) suggest that 12 h after treatment, the quantity of migrated cells was higher in the hucMSCs-EX or hucMSCs-CM group in contrast to the control group.

### 3.5 hucMSCs-EX Induces M2 Macrophage Phenotype by Suppressing the LPS-induced Stimulation of NF-κB p65 and STAT3 Pathways

**Fig. (5):**
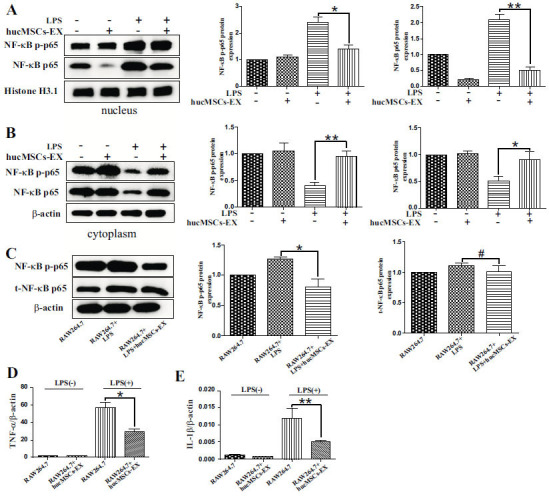
hucMSCs-EX inhibits LPS-induced NF-κB P65 stimulation. **(A), (B),** Cells were treated LPS (1μg/mL) for 2 h and then incubated with hucMSCs-EX for 48 h. The WB analysis determined the level of NF-κB P65 (nucleus) and NF-κB p-P65 (cytoplasm) in RAW264.7 cells. Both β-actin and H3.1 were employed as reference controls for cell cytoplasmic and nuclear proteins **(C)**, hucMSCs-EX inhibited the phosphorylation of NF-κB p65 (NF-κB p-p65) in respective cells stimulated by LPS. **(D), (E)** Cells were treated with LPS (1μg/mL) for 4 h, then maintained in hucMSCs-EX for 48 h. The mRNA expressions of *IL-1β* and *TNF-α* were assessed *via* RT-PCR. (*p ≤ 0.05, ** *p* ≤ 0.01, # *p* ≥ 0.05;).

**Fig (6):**
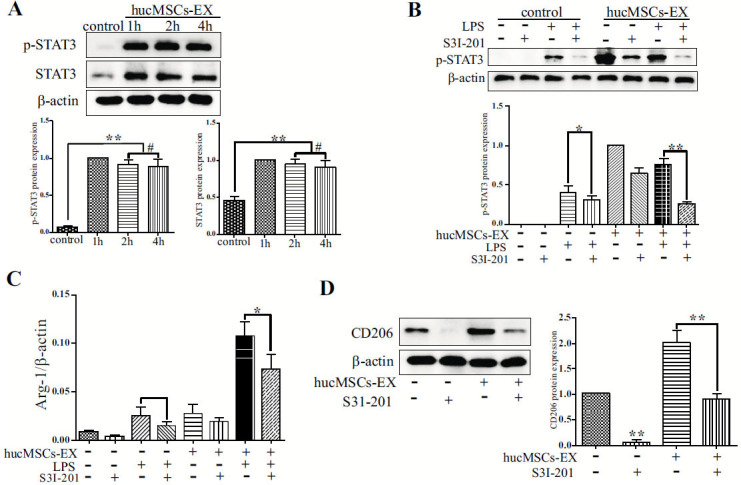
hucMSCs-EX induces M2 polarization *via* STAT3 pathway stimulation. **(A),** Cells were maintained for 1, 2, and 4 h in hucMSCs-EX or control medium. The p-STAT3 and STAT3 concentrations were measured *via* WB analysis. Protein levels of STAT3 and p-STAT3 were markedly increased by hucMSCs-EX (**p ≤ 0.01, # *p* ≥ 0.05). **(B),** Cells were incubated with hucMSCs-EX or a control medium for 24 h with or without S3I-201 (100 μmol/L). The STAT3 phosphorylation of cells was then confirmed by WB analysis after 2 h of incubation with LPS (1 μg/mL) (*p ≤ 0.05, ** *p* ≤ 0.01). **(C),** The content of RNA was isolated from the RAW264.7 cells (B), and the *Arg-1* mRNA was amplified by qRT-PCR (*p ≤ 0.05). **(D),** Cells were grown in hucMSCs-EX or complete medium for 24 h, with or without S3I-201 (100 μmol/L). The quantification of CD206 expression in respective cells was carried out *via* WB analysis (**p ≤ 0.01).

Western blotting determined the concentrations of the cytoplasmic protein NF-κB p-p65 and the nuclear protein NF-κB P65 in respective cells. As illustrated in Fig. (**5A** and **B**), the distribution of NF-κB p65 proteins in uninduced RAW 264.7 cells was predominantly found in the cytoplasmic fraction. An increase in phosphorylated NF-κB p65 (NF-κB p-p65) and NF-κB p65 was seen after 1 h of LPS stimulation (Fig. **5A**). However, this effect was antagonized by hucMSCs-EX (Fig. **5A** and **C**). The qRT-PCR findings indicated that the NF-κB p65 activation induced by LPS specifically targeted *IL-1β* and *TNF-α* (Fig. **5D** and **E**). The expressions of *TNF-α* (Fig. **5D**) and *IL-1β* (Fig. **5E**) were inhibited by hucMSCs-EX. The results demonstrated that hucMSCs-EX inhibited the LPS-promoted phosphorylation of NF-κB p65, NF-κB p65, and the downstream secretions of *TNF-α* and *IL-1β*. Considering the significance of the NF-κB and STAT3 pathways in M2 macrophage polarization [21], a possible connection between the role of MSCs-EX and STAT3 signaling was monitored. RAW264.7 cells were exposed to hucMSCs-EX at various time intervals. The WB analysis revealed a significant upregulation of p-STAT3 expression 1 h after treatment, which persisted in an activated state for the next 4 h (Fig. **6A**). To confirm the function of STAT3 stimulation in the polarization induced by hucMSCs-EX, cells were treated with hucMSCs-EX with or without the presence of STAT3 inhibitor, S3I-201, under both normal and LPS-induced environments. The findings indicated that the upregulation of Arg-1 and the activation of p-STAT3 by hucMSCs-EX were both inhibited by S3I-201 (Fig. **6B** and **C**). Further, S3I-201 inhibited the synthesis of CD206 protein in cells induced by hucMSCs-EX (Fig. **6D**). Collectively, these results demonstrate that MSCs-EX stimulates the STAT3 pathway in respective cells to induce M2 polarization.

## 4 DISCUSSION

Macrophages can be classified into M1 or M2 phenotypes based on their environment. They have significant functions in initiating and advancing inflammatory responses [22]. M1 macrophages are identified by the high expression of *IL-6, TNF-α, IL-8*, and *IL-12,* whereas M2 macrophages are recognized by the high levels of *Arg-1, IL-10,* and *CD206*. Studies have indicated that hucMSCs-CM have potent chemoattractive effects on monocytes *in vitro* and promote macrophage proliferation during wound healing mechanism [14, 23]. Dayan *et al.* [24] revealed that acute myocardial infarction mice infused with MSCs had a substantial increase in the macrophage numbers in the heart and circulation, which could be linked with improved cardiac function. The findings of this study suggested that hucMSCs-EX induced migration and proliferation of RAW264.7 cells *in vitro*, despite LPS stimulation and confirm previous reports that suggested that exosomes derived from MSCs may affect inflammatory effects by recruiting macrophages against inflammatory stimuli. This study evaluated markers associated with the M1 and M2 activation stages and indicated that hucMSCs-EX can prevent the LPS-induced polarization of respective cells into M1-type macrophages. Moreover, hucMSCs-EX also stimulated the differentiation of RAW264.7 cells into the M2 phenotype. These results indicate that exosomes derived from MSCs could induce a phenotype switch of the recruited macrophages in the inflammatory micro-environment to favor relief of inflammation. However, more studies are required to understand the precise mechanism through which hucMSCs-EX affects the polarization of macrophages in the inflammatory response.

Recent research has demonstrated that NF-κB regulates macrophage polarization, a crucial factor in determining whether inflammatory events initiate or terminate [25]. Moreover, they have also found that the p65 (RelA) is a vital member of the NF-κB family that contributes to the M1 macrophage polarization and the secretion of inflammatory mediators [7, 25, 26]. The current study revealed that NF-κB p65 nuclear translocation was impeded by MSCs-EX against LPS induction which could help to describe the downregulation of M1 macrophage-associated genes after treatment with hucMSCs-EX. According to Qian *et al.* [27], the activation of STAT3 is linked to the macrophage M2 phenotype and the release of anti-inflammatory factors. Moreover, STAT3 has a crucial function in preserving the equilibrium between pro-inflammatory and anti-inflammatory factors in immune-competent cells, thereby maintaining homeostasis. The impact of MSCs-EX on STAT3 in respective cells was examined. It was observed that the levels of phosphorylated STAT3, Arg-1, and CD206 in cells were markedly raised by hucMSCs-EX. It was also found that the STAT3 inhibitor S3I-201 effectively counteracted the phosphorylation of STAT3 and the expression of Arg-1 and CD206 induced by hucMSCS-EX in cells. Based on these findings, phosphorylated STAT3 was implicated in the polarization of macrophage M2 induced by MSCs-EX, whereas LPS-induced macrophage M1 polarization engaged NF-κB p65, which could potentially be inhibited by MSCs-EX. Overall, the induction of the M2 phenotype in macrophages was promoted by MSCs-EX *via* the stimulation of the STAT3 and the suppression of NF-κB pathways.

This study suggests that hucMSCs-EX induced the activation of STAT3 pathways and inhibited the stimulation of the NF-κB p65 pathway, thereby promoting macrophages to adopt an M2 phenotype. This led to the formation of an anti-inflammatory microenvironment. Moreover, the anti-inflammatory mechanism was identified as one by which hucMSCs-EX inhibits infection and tissue damage. Tissue repair could potentially be stimulated through the efficient regulation of inflammation, a universal response against injury. The potential therapeutic application of hucMSCs-EX stems from their ability to promote tissue repair *via* modulation of inflammatory-associated immune cells.

## CONCLUSION

In conclusion, this study presented an alternate approach for controlling the inflammatory response using MSC-derived exosomes as mediators, which may direct macrophages toward the M2 phenotype. Therefore, our study provides a novel perspective for improving MSC functions in clinical applications by regulating inflammatory responses.

## Figures and Tables

**Table 1:** Sequences of specific primers used for gene expression analysis.

**Genes**	**Sequence (5’-3’)**	**Length, nt**	**Size (bp)**	**Annealing Temperature, (°C)**
TNF-α	For: AACTCCAGGCGGTGCCTATG Rev: TCCAGCTGCTCCTCCACTTG	20 20	242	64
IL-6	For: AAGTCCGGAGAGGAGACTTC Rev: TGGATGGTCTTGGTCCTTAG	20 20	487	58
IL-10	For ACTCTTCACCTGCTCCACTG Rev: GCTATGCTGCCTGCTCTTAC	20 20	415	60
Arg-1	For: CCAGATGTACCAGGATTCTC Rev: AGCAGGTAGCTGAAGGTCTC	20 20	191	55
IL-1β	For: AGCTTCAGGCAGGCAGTATC Rev: TCATCTCGGAGCCTGTAGTG	20 20	215	60
β-actin	For: CACGAAACTACCTTCAACTCC Rev: CATACTCCTGCTTGCTGATC	21 20	265	55

## Data Availability

We declare that all data and supporting information is provided in this article.

## LIST OF ABBREVIATIONS

MSCs: Mesenchymal Stem Cells
NKCs: Natural Killer Cells
WB: Western Blot
